# Identification of Circulating Gene Expression Signatures of Intracranial Aneurysm in Peripheral Blood Mononuclear Cells

**DOI:** 10.3390/diagnostics11061092

**Published:** 2021-06-15

**Authors:** Vincent M. Tutino, Haley R. Zebraski, Hamidreza Rajabzadeh-Oghaz, Muhammad Waqas, James N. Jarvis, Konrad Bach, Maxim Mokin, Kenneth V. Snyder, Adnan H. Siddiqui, Kerry E. Poppenberg

**Affiliations:** 1 Canon Stroke and Vascular Research Center, University at Buffalo, Buffalo, NY 14203, USA; hrajabza@buffalo.edu (H.R.-O.); mwaqas@ubns.com (M.W.); ksnyder@ubns.com (K.V.S.); asiddiqui@ubns.com (A.H.S.); kerrypop@buffalo.edu (K.E.P.); 2 Department of Pathology and Anatomical Sciences, University at Buffalo, Buffalo, NY 14203, USA; 3 Department of Neurosurgery, University at Buffalo, Buffalo, NY 14203, USA; 4 Department of Mechanical and Aerospace Engineering, University at Buffalo, Buffalo, NY 14228, USA; 5 Department of Biomedical Engineering, University at Buffalo, Buffalo, NY 14228, USA; haleyzeb@buffalo.edu; 6 Department of Pediatrics, University at Buffalo, Buffalo, NY 14203, USA; jamesjar@buffalo.edu; 7 Department of Neurosurgery and Brain Repair, University of South Florida, Tampa, FL 33620, USA; kbach@usf.edu (K.B.); mokin@usf.edu (M.M.)

**Keywords:** cerebral aneurysm, monocytes, T lymphocytes, transcriptome profiling, biomarkers

## Abstract

Peripheral blood mononuclear cells (PBMCs) play an important role in the inflammation that accompanies intracranial aneurysm (IA) pathophysiology. We hypothesized that PBMCs have different transcriptional profiles in patients harboring IAs as compared to IA-free controls, which could be the basis for potential blood-based biomarkers for the disease. To test this, we isolated PBMC RNA from whole blood of 52 subjects (24 with IA, 28 without) and performed next-generation RNA sequencing to obtain their transcriptomes. In a randomly assigned discovery cohort of *n* = 39 patients, we performed differential expression analysis to define an IA-associated signature of 54 genes (*q* < 0.05 and an absolute fold-change ≥ 1.3). In the withheld validation dataset, these genes could delineate patients with IAs from controls, as the majority of them still had the same direction of expression difference. Bioinformatics analyses by gene ontology enrichment analysis and Ingenuity Pathway Analysis (IPA) demonstrated enrichment of structural regulation processes, intracellular signaling function, regulation of ion transport, and cell adhesion. IPA analysis showed that these processes were likely coordinated through NF-kB, cytokine signaling, growth factors, and TNF activity. Correlation analysis with aneurysm size and risk assessment metrics showed that 4/54 genes were associated with rupture risk. These findings highlight the potential to develop predictive biomarkers from PBMCs to identify patients harboring IAs.

## 1. Introduction

Intracranial aneurysms (IAs) are pathological outpouchings within cerebral vasculature that are present in about 3–6% of the general population [1,2]. The rupture of an IA, which is the predominant cause of non-traumatic subarachnoid hemorrhage (SAH), is associated with high mortality and morbidity rates [3,4]. Since most IAs are asymptomatic until rupture, unruptured aneurysms are typically detected incidentally by cerebral imaging performed for other reasons. While early IA detection can allow for closer monitoring and preventative treatments, imaging for general IA screening is unsuitable due to high costs and potential risks [5]. Recently, RNA profiling of circulating immune cells has emerged as a potential means to identify robust diagnostic markers of vascular diseases, such as IA, aortic aneurysms, and stroke [6,7,8,9,10,11].

Clinical reports and animal model studies have shown that IA pathology is dominated by inflammation [1,12,13,14]. The initial inflammatory responses during aneurysm genesis are thought to be perpetuated by the endothelium and pro-inflammatory vascular smooth muscle cells [15,16]. During IA natural history, chemokines and cytokines released into the blood by the diseased tissue may begin to recruit circulating immune cells to the lesion [17]. Reports have shown increased neutrophil-to-lymphocyte ratios in patients with larger, more progressed IAs, and with ruptured aneurysms, suggesting a role for peripheral activation of immune cells and systemic innate inflammatory responses during IA [18,19]. Indeed, circulating monocytes, neutrophils, and other lymphocytes in the blood are known to infiltrate the nascent IA wall and contribute to maladaptive remodeling via upregulation and release of matrix metalloproteinases (MMPs) [20,21]. Traditionally, macrophages have been recognized as the main contributor to inflammation in IA [22,23]. Studies of both ruptured and unruptured IA tissue have reported increased presence of T cells, B cells, and macrophages within the vascular walls of IAs, and even more so in ruptured ones [24,25]. Furthermore, animal models have demonstrated that both the depletion of macrophages and the inhibition of monocyte chemoattractants are associated with a reduced aneurysm formation [26,27].

In this study, we hypothesized that peripheral blood mononuclear cells (PBMCs), which are predominantly composed of monocytes, T cells, and B cells, have different transcriptional profiles in patients with IAs as compared to IA-free patients. Our rationale was that ongoing inflammatory responses at the IA tissue level could lead to peripheral cellular activation and chemoattraction of PBMCs, which has also been observed for circulating neutrophils in IA. Sabatino et al. demonstrated the initial feasibility of this concept in 2013 [28]. Using a cohort of 15 ruptured IAs, 15 unruptured IAs, and 15 controls, they performed gene chip microarrays on PBMC RNA and found over expression of pro-apoptotic genes and under expression of extracellular matrix-related genes were associated with IA [28]. Yet, while they provided important preliminary evidence of potential PMBC expression signatures of IA, the previous efforts investigated small datasets, did not include validation studies, and used microarray technology that is less sensitive and comprehensive than modern RNA sequencing techniques.

To overcome these challenges and test our hypothesis, we performed transcriptome profiling on PBMCs in patients with and without IAs (confirmed on angiography). We used next-generation RNA sequencing to identify an IA-associated expression signature in PBMC transcriptomes. We further assessed if the IA-associated signature could distinguish patients with and without IA in a small, independent cohort of patients. Gene ontology analysis and physiological pathway modeling were used to determine the biological function of differentially expressed transcripts. Results from this study could motivate future efforts towards developing blood-based biomarkers and shed light on the pathophysiology of aneurysms.

## 2. Materials and Methods

### 2.1. Patient Enrollment and Cohort Creation

This study was approved by the University at Buffalo’s Human Research Institutional Review Board (study numbers 030474433 approved 8/20/2013-5/19/2022 and 00005225, approved 3/16/2021-3/15/2022). Written informed consent was obtained from all subjects prior to sample collection and the study was carried out in accordance with the approved protocol. Patients at Gates Vascular Institute (Buffalo, NY, USA) receiving cerebral digital subtraction angiography (DSA) with and without IA diagnosis were enrolled in this study. Indications for DSA included confirmation of IAs detected on noninvasive imaging or follow-up imaging of previously detected IAs for the IA group, or to identify presence or absence of vascular disease (i.e., malformations, carotid stenosis) for the control group. Patients who consented to participate in this study were over 18 years old, English-speaking, and had not previously been treated for IA. Patients who were pregnant, had a fever (>100 °F), recently had invasive surgery, were receiving chemotherapy treatments, had autoimmune disease, or were on immunomodulating drugs were excluded. Information about patient’s history and comorbidities was collected from medical records.

Between December 2013 and September 2018, we collected whole blood samples from patients receiving cerebral DSA (103 with IA, 129 without IA) [6,7,8,29]. Before sequencing, samples were further screened by demographics and comorbidities. To control for potential confounding variables, samples were removed if they had more than 3 comorbidities (including smoking, hypertension, heart disease, diabetes mellitus, osteoarthritis, stroke history, and asthma) or any other cerebral vascular disease. Prior to data analysis, samples were divided into a discovery and validation cohort using a 75%:25% split, such that the expression of identified differentially expressed genes could be independently verified.

### 2.2. PBMC Isolation and RNA Preparation

Whole blood was drawn into 10-mL citrated CPT tubes (Becton Dickinson, Franklin Lakes, NJ, USA) from the femoral access sheath. Cell separation procedures occurred within 1 h from when the specimens were drawn. PBMCs were separated from granulocytes and red blood cells (RBCs) by density gradient centrifugation. The PBMCs were washed with RBC lysis buffer, pelleted and then disrupted with TRIzol reagent (Invitrogen, Carlsbad, CA, USA) and stored at −80 °C until further processing. RNA was extracted using the TRIzol kit, according to the manufacturer’s instructions. Trace amounts of DNA were removed by DNase I (Life Technologies, Carlsbad, CA, USA) treatment. RNA was purified using the RNeasy MinElute Cleanup Kit (Qiagen, Venlo, Limburg, Netherlands) and suspended in RNase-free water. The purity and concentration of RNA in each sample were measured by absorbance at 260 nm on a NanoDrop 2000 spectrophotometer (Thermo Scientific, Waltham, MA, USA), and 400 ng of RNA was sent to the University at Buffalo’s Next-Generation Sequencing and Expression Analysis Core facility for further quality control. Precise RNA concentration was measured at the core facility via the Quant-iT RiboGreen Assay (Invitrogen, Carlsbad, CA, USA) with a TBS-380 Fluorometer (Promega, Madison, WI, USA). The quality of the RNA samples was measured with an Agilent 2100 BioAnalyzer RNA 6000 Pico Chip (Agilent, Las Vegas, NV, USA). RNA samples of acceptable purity (260/280 ratio of ≥1.9) and integrity (RQN  ≥  8.0) were considered for RNA sequencing.

### 2.3. RNA Sequencing

RNA libraries were prepared using the Illumina TruSeq stranded total RNA gold kit (Illumina, San Diego, CA, USA). All samples underwent 100-cycle, dual-read sequencing in the Illumina NovaSeq6000 System (Illumina, San Diego, CA, USA) on a single flow cell and were demultiplexed with Bcl2Fastq. Per-cycle basecall files generated by the NovaSeq6000 were converted to pre-read FASTQ files using bclfastq version 2.20.0.422 using default parameters. The quality of the sequencing was reviewed using FastQC version 0.11.5. Potential contamination was detected using FastQ Screen version 0.11.1. No adapter sequences were detected, so no trimming was performed. Genomic alignments were performed using HISAT2 version 2.1.0 using default parameters. NCBI reference GRCh38 was used for the reference genome and gene annotation set. Sequence alignments were compressed and sorted into binary alignment map files using samtools version 1.3. Mapped reads for genomic features were counted using Subread featureCounts version 1.6.2 using the parameters -s 2 –g gene_id–t exon–Q 60; the annotation file specified with —a was the NCBI GRCh38 reference from Illumina iGenomes [30,31,32].

### 2.4. Differential Expression Analysis

For differential expression analysis raw counts were normalized as transcripts per million (TPM). Protein coding genes with expression in >50% of all samples were considered for differential expression analysis using an F-test. Those with a John Storey False Discovery Rate (FDR) corrected *p*-value (*q*-value) < 0.05 and with an absolute fold change ≥ 1.3 were considered differentially expressed. To visualize how differentially expressed genes (DEGs) could separate control and IA samples, we performed hierarchical clustering (complete linkage, Euclidean distance) on scaled log-transformed data and visualized the results in a heatmap. We also performed *k*-means clustering using 1-pearson correlation metric, *k* = 2, and 1000 iterations (https://software.broadinstitute.org/morpheus/, accessed 22 April 2021).

Cell composition analysis was performed using the open-access CIBERSORT application (version 1.06, https://cibersort.stanford.edu/, accessed on 22 April 2021) [33]. Analysis was performed on TPM-normalized gene expression values and considered 22 leukocyte cell-type signatures. CIBERSORT used a linear support vector regression to estimate cell proportions. Protein coding transcripts with expression in >50% of all samples were used in this analysis (*n* = 9239). A Student’s *t*-test was used to evaluate if there were any significant differences in predicted cell populations between the IA and control groups (*q*-value < 0.05 was considered significant).

### 2.5. Bioinformatics

Based on our differential expression analysis, we studied ontological enrichment in our significantly differentially expressed genes using the g:GOSt tool (https://biit.cs.ut.ee/gprofiler/gost, accessed on 22 April 2021) in g:Profiler program [34,35], using all knowns transcripts as background. This enabled us to identify enriched biological process, molecular function, and cellular component GO terms in genes with increased or decreased expression. Reported ontologies with 5 or more focus molecules, along with their FDR-adjusted *p*-values, were summarized and visualized as networks in the REVIGO (REduce and VIsualize Gene Ontology, http://revigo.irb.hr/, accessed on 22 April 2021) tool [36] using a semantic similarity cutoff of C = 0.70 (only ontologies with at least one other network connection were retained for visualization).

Additional analyses were performed in Ingenuity Pathway Analysis (IPA, Qiagen QIAGEN Inc., https://www.qiagenbioinformatics.com/products/ingenuitypathway-analysis, accessed on 22 April 2021) [37]. Here, IPA was used to generate networks of potential interactions. For IPA, each gene identifier was mapped to its corresponding gene object in the Ingenuity Knowledge Base and overlaid onto a molecular network derived from information accumulated in the Knowledge Base. Gene networks were algorithmically generated based on their “connectivity” derived from known interactions between the products of these genes. Networks with *p*-scores > 20 were considered significant. We also assessed predicted upstream regulators in IPA. Biomolecules (RNA, protein, DNA) with a Benjamini–Hochberg FDR-corrected *p*-value < 0.05 were considered regardless of z-score.

### 2.6. The Differentially Expressed Genes in the Validation Cohort

To determine if differentially expressed genes could also separate patients with IAs from controls in an independent cohort, we evaluated their expression in the validation cohort that consisted of 25% of all samples. For comparison, we calculated their fold-change in this new cohort. Principal component analysis using the prcomp package in R was used to determine if the genes could delineate IA from controls in both cohorts. To further explore their diagnostic potential, we performed supervised machine learning on the TPM data using the MATLAB Statistics and Machine Learning Toolbox (MathWorks, Natick, MA, USA). In the discovery dataset, we trained a Subspace Discriminant Ensemble model with four-fold cross-validation to avoid overfitting. This model was then tested in the validation dataset. In training and testing, we calculated the model’s accuracy, sensitivity, and specificity.

### 2.7. IA Risk Correlation

As aberrant RNA expression in circulating PBMCs may be aggravated in cases of larger or more developed IAs, we explored the relationship between expression of DEGs and IA risk. We assessed risk in two ways: (A) By measuring IA size (the most common clinical metric for assessing IA risk) and (B) By calculating 5-year rupture risk % according to the International Study of Unruptured Intracranial Aneurysms (ISUIA) study [38]. To identify genes correlated with each risk classification, we implemented Pearson correlation analysis. The degree of correlation was assessed using the Pearson correlation coefficient (PCC) and *p*-values from the Wald test (α = 0.05). A “very strong” correlation was defined by 1.0 ≥ |PCC| ≥ 0.80, 0.79 ≥ |PCC| ≥ 0.60 defined “strong” correlation, 0.59 ≥ |PCC| ≥ 0.40 defined “moderate” correlation, 0.39 ≥ |PCC| ≥ 0.20 defined “weak” correlation, and |PCC| < 0.19 defined a “very weak” or no correlation [39].

## 3. Results

### 3.1. Study Population

We studied the gene expression profiles from a total of 52 peripheral blood samples (24 IA, 28 controls) that met our inclusion and quality criteria. As shown in Table 1, our cohort matching scheme resulted in a discovery cohort population in which there were no statistically significant differences in age, sex, smoking, and comorbidities between the two groups in the discovery cohort of 39 patients. Characteristics of the IAs in the discovery group are demonstrated in Supplementary Material Table S1. In this group, aneurysm size (largest diameter measured on DSA) ranged from 2 to 18 mm, with a mean size of 6.2 mm. Furthermore, one individual had multiple IAs, and three people had a reported family history of aneurysm.

We performed RNA sequencing to identify differentially expressed genes in PBMCs. The RNA quality and sequencing quality metrics for all samples are reported in Supplemental Tables S2 and S3. The 52 sequenced samples had an average 260/280 of 2.09 and an average RNA quality number of 9.40. On average, there were 32.2 million reads assigned per sample, and a 96.3% aligned rate. To determine if differentially expressed transcripts were related to presence of IA, rather than differences in cell populations, we estimated the proportions of different cell populations in each sample using CIBERSORT. This analysis showed no statistically significant difference in proportions of cell types between all control and IA samples in the discovery cohort (all *q* > 0.05). On average, monocytes represent the majority (29%) followed by CD4+ T cells (28%), NK cells (18%), CD8+ T cells (12%), B cells (4%), Tregs (3%), neutrophils (3%—likely a contaminant from processing), mast cells (1%), and macrophages (1%) (see Supplemental Figure S1).

### 3.2. Differentially Expressed Genes in PBMCs from Patients with IA

**Figure 1:**
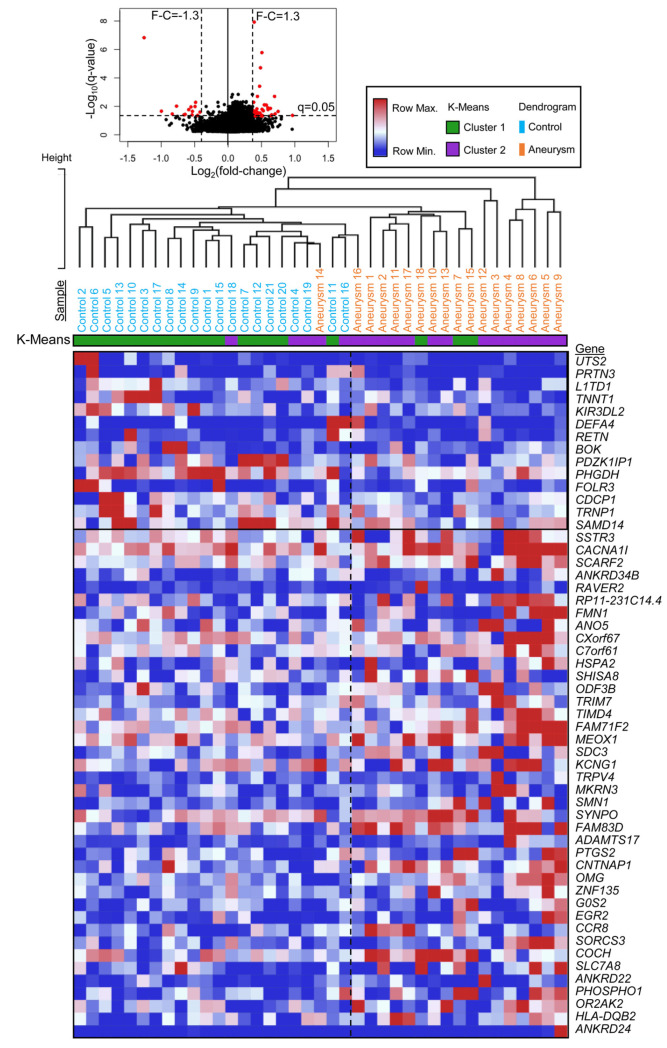
**Differential gene expression analysis.** The volcano plot at the top demonstrates differential RNA expression between the two groups. Red circles indicate an IA-associated signature of significantly differentially expressed transcripts (*q* < 0.05) with an absolute fold-change ≥ 1.3. The heatmap below also shows differential expression of the 54 genes with increasing fold-change (descending). Hierarchical clustering using these 54 genes was able to separate the control (blue) and IA (orange) samples well; K-means clustering showed 85% (33/39) of the samples were clustered their respective group (green with control, purple with IA). (Abbreviations: F-C = fold-change, Max. = maximum, Min. = minimum).

The volcano plot in Figure 1 shows PBMC expression differences between the IA patients and control subjects of the discovery cohort in terms of average fold-change in expression and significance level. We identified 54 genes from 9239 protein coding transcripts with testable expression (expression in >50% of all samples) that were significantly differentially expressed (*q* < 0.05 and an absolute fold-change ≥ 1.3) between the two groups of the discovery cohort, reported in Table 2. Fourteen genes had lower expression in IA, and 40 genes had higher expression in IA. These genes and how they were able to cluster the two cohorts are shown in the heatmap in Figure 1. Furthermore, K-means clustering using this signature was able to correctly assign 85% (33/39) of the samples to their respective group in the discovery cohort.

### 3.3. Bioinformatics Analyses

**Figure 2:**
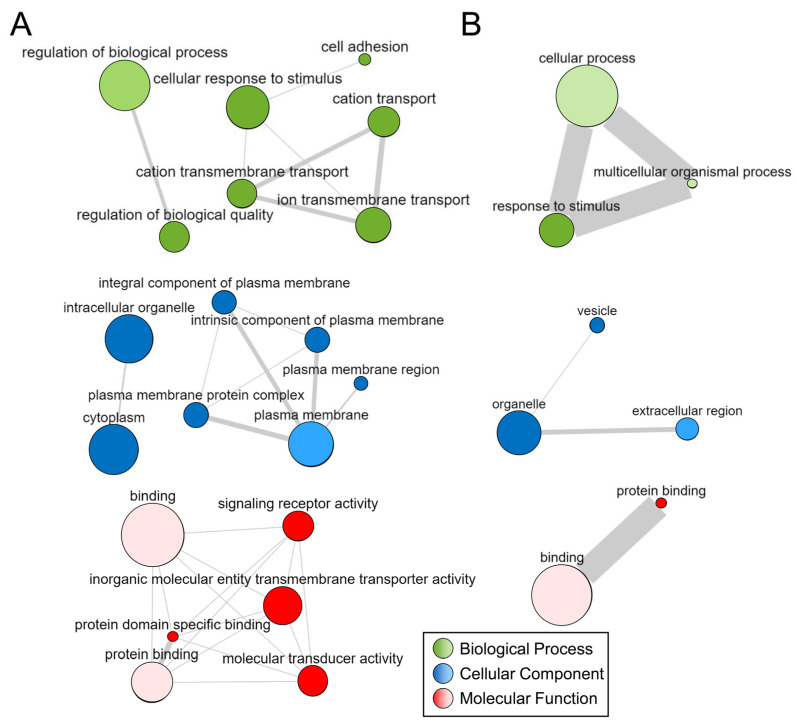
**Networks derived from significantly enriched ontologies.** The figure shows relationships of biological process (green), cellular component (blue), and molecular function (red) gene ontologies as visualized by the interactive graph module in REVIGO. Here, ontologies with greater significance (*q*-value) are larger and similar terms are linked by bars whose width indicates the degree of similarity (ontologies without links are not visualized here). (**A**) Enriched ontologies in significant genes with higher expression in IA. (**B**) Enriched ontologies in significant genes with lower expression in IA. (Abbreviations: REVIGO = REduce and VIsualize Gene Ontology).

To understand the biological implication of differential PBMC RNA expression, we performed several bioinformatics analyses. We performed detailed gene ontology term enrichment analysis using g:Profiler, then reduced and visualized GO terms using REVIGO. Networks of significant GO terms for the significant up- and down-regulated genes are shown in Figure 2. From this analysis, upregulated genes showed enrichment of terms related to adhesion processes, ion transport and structural processes, transmembrane and junction components, and signaling receptor activity. On the other hand, downregulated genes were enriched for terms related to response to stimulus processes, vesicle components, and binding activity function (a full list of ontologies is reported in Supplemental Table S4). Overall, these functions are pertinent to monocyte activation and recruitment via intravascular perturbations, as they reflect processes related to response to stimuli, cellular adhesion, structural reorganization, and cell-to-cell signaling.

**Figure 3:**
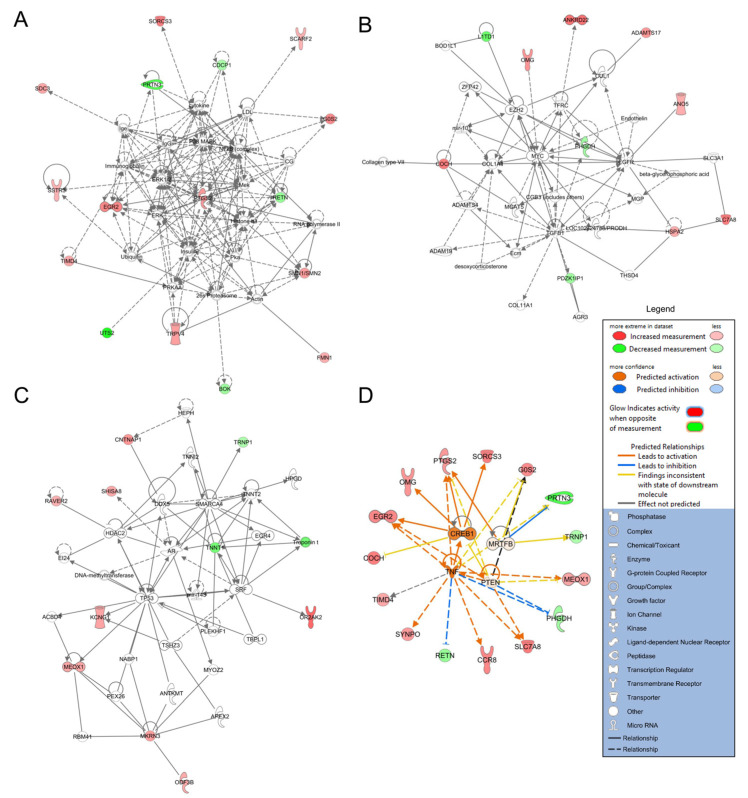
**Ingenuity Pathway Analysis results.** Networks and upstream regulators were derived from IPA using differentially expressed genes in PBMCs from IA patients and controls. Genes with increased expression levels in patients with IAs are red, and genes with lower expression levels in patients with IAs are green, while fold-change is represented by color intensity. Non-differentially expressed transcripts with known interactions are not colored. Direct and indirect relationships are shown by solid and dashed lines, respectively. (**A**) The first network (*p*-score = 36) shows nodes of interactions to differentially expressed genes at NF-kB, PTGS2 (also differentially expressed), cytokines, and immunoglobulins. (**B**) The second network (*p*-score = 20) shows nodes of regulation at TGFB, MYC, and PHGDH, as well as other interactions with ADAMTS family members and collagens. (**C**) The third network (*p*-score = 20) shows nodes of regulation at TP53, SRF, and SMARCA4. (**D**) Predicted upstream regulators are shown in orange (activation) or blue (inhibition). This network of significant upstream regulators shows evidence that TNF and CREB1 may have a role in regulating predominantly genes with increased expression.

All 54 significantly differentially expressed genes were also analyzed using IPA. As demonstrated in Figure 3, IPA revealed three significant networks (*p*-score > 20) relating to the significant genes. The first network (Figure 3A) had a *p*-score of 36 and was associated with “behavior”, “cell death and survival”, and “connective tissue disorders”, with nodes consisting of inflammatory signaling molecules, such as the transcription factor NF-kB, cytokines, and immunoglobulins (IgG). Network 2 (Figure 3B) had a *p*-score of 20. It was associated with “amino acid metabolism”, “cell cycle”, and “cellular development” processes, and had nodes at extracellular matrix components such as ADAMTS genes, and collagens, as well as the growth factors TGFB and EGFR. Lastly, Network 3 (Figure 3C) also had a *p*-score of 20, and was enriched for “cardiovascular system development and function”, “cellular assembly and organization”, and “cellular development” ontologies. This network was related to genes that extend the lifecycle of cells, with nodes at TP53, SRF, and SMARCA4. Figure 3D shows that potential upstream regulators of gene expression identified in IPA included activation by TNF and CREB1 (*q* < 0.05, albeit their z-scores were not greater than 2.0). See Supplemental Tables S5 and S6 for additional data on the networks and upstream regulators, respectively.

### 3.4. Replication of Fold-Change in the Validation Cohort

**Figure 4:**
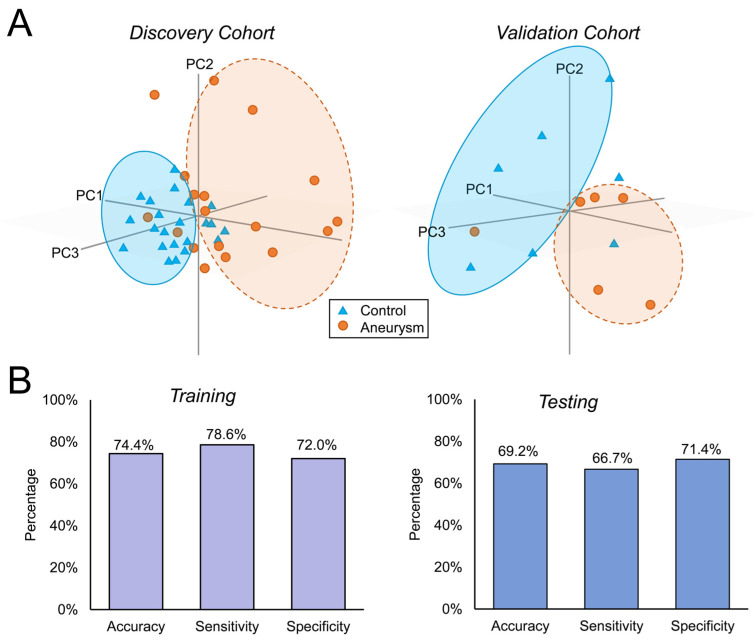
**Replication study in a validation cohort of 13 patients (6 with IAs).** (**A**) The two plots show principal component analysis performed using the 54 IA-associated genes in both the discovery (left) and validation (right) cohorts. The gene set is able to distinguish the IA samples (orange circles) from the controls (blue triangles) in a similar fashion. (**B**) Supervised machine learning was performed to determine how well the 54 genes delineated the IA from control samples in both datasets. The bar graph on the left shows that training an Ensemble classifier in the discovery cohort yielded a mean cross-validation accuracy, sensitivity, and specificity ≥72%. In testing the validation dataset, our data shows that the model still had moderate performance, with an accuracy = 69.2%, a sensitivity = 66.7%, and a specificity = 71.4%.

To determine whether expression of the IA-associated signature could separate patients with IAs from controls in an independent cohort, we performed a small replication study in the validation cohort of *n* = 13 patients (see Supplemental Tables S7 and S8 for this cohort’s demographic/comorbidity data and aneurysm characteristics, respectively). Patients with IAs (*n* = 6) had aneurysms ranging in size from 3 to 10 mm (average size = 6.4 mm) and included one individual with multiple aneurysms. TPM levels from RNA sequencing data for the 54 genes of interest were used to visualize how these transcripts could distinguish the IA group from the control group. PCA using these genes in both the discovery and validation cohort are shown in Figure 4A. This analysis demonstrated that in both cohorts, the 54 genes could well separate the IAs from controls in the principal component space. This may be because over half of the 54 genes (30 genes, 56%) displayed the same direction fold-change in expression in the validation cohort (see Supplemental Figure S2). Additionally, our supervised machine learning analysis also demonstrated that the 54 genes could help delineate IA from control samples. The Ensemble classifier, trained in the discovery dataset (accuracy = 74.4%, sensitivity = 78.6%, and specificity = 72.0%), was able to identify IA cases in the validation cohort with moderate accuracy = 69.2%, a sensitivity = 66.7%, and a specificity = 71.4% (Figure 4B).

### 3.5. Correlation of Gene Expression with IA Risk

**Figure 5:**
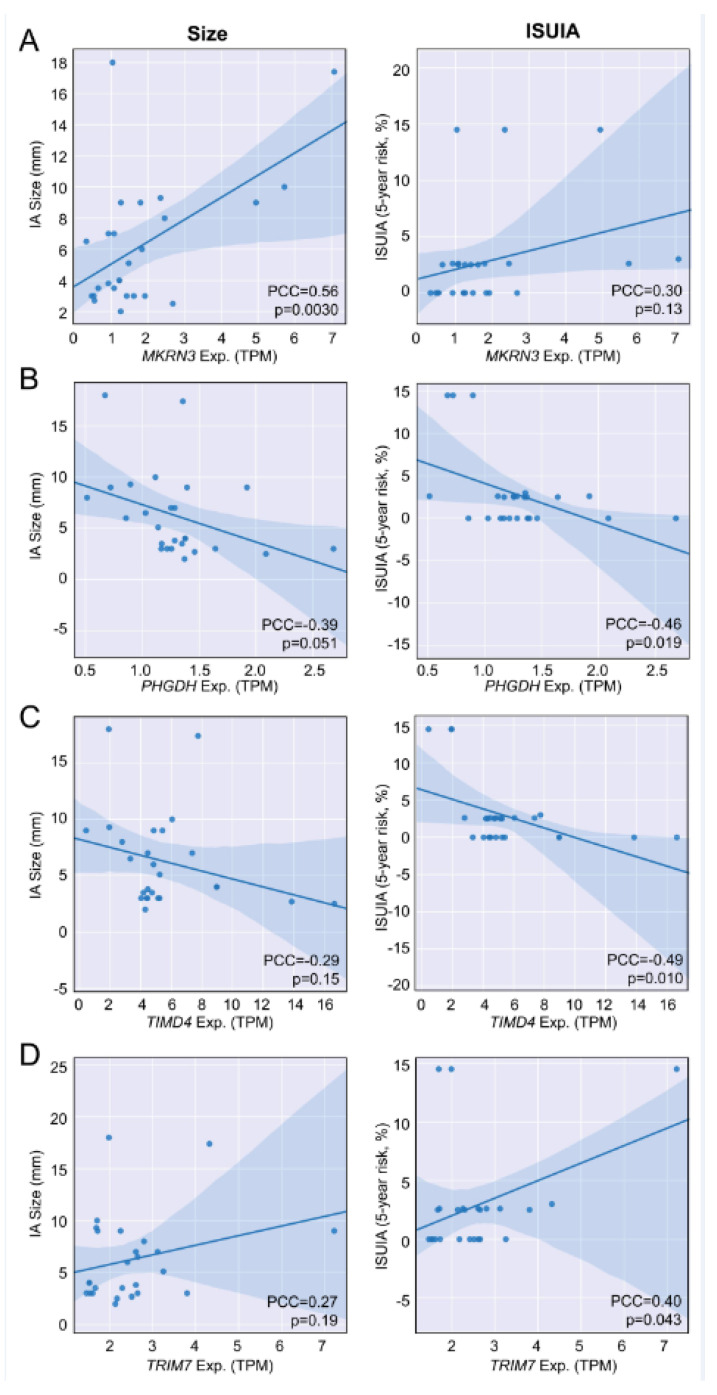
**Genes significantly correlated with the IA risk metrics, IA size and ISUIA-defined 5-year rupture risk %.** Four genes had an absolute PCC > 0.25 in both correlation analyses, with at least one correlation being significant (*p* < 0.05). (**A**) *MKRN3* expression was most significantly, positively correlated with IA size (PCC = 0.56, “moderate”). (**B**) *PHGDH* expression was most significantly, negatively correlated with 5-year risk (PCC = −0.46, “moderate”). (**C**) *TIMD4* expression was also most significantly, negatively correlated with 5-year risk (PCC = −0.49, “moderate”). (**D**) TRIM7 expression was most significantly, positively correlated with 5-year risk (PCC = 0.40, “moderate”). (Exp. = expression, ISUIA = International Study of Unruptured Intracranial Aneurysms, PCC = Pearson correlation coefficient, TPM = transcripts per million).

We used Pearson correlation analysis to explore the relationship between the expression of the 54 significant genes and IA risk, as measured by aneurysm size and 5-year rupture risk % (from ISUIA). As shown in Supplemental Table S9, three genes had significant correlation to size (all had a |PCC| > 0.40), and five had significant correlation to 5-year rupture risk (again, all had a |PCC| > 0.40). Figure 5 shows the correlation plots for size and ISUIA of four genes (*MKRN3*—Figure 5A, *PHGDH*—Figure 5B, *TIMD4*—Figure 5C and *TRIM7*—Figure 5D) that had an absolute PCC > 0.25 for both risk assessments. Based on this analysis, these genes may be good candidates as biomarkers for IA rupture risk in addition to IA presence.

## 4. Discussion

It is widely known that the natural history of IA is accompanied by progressive inflammatory responses in the vascular wall [1,12,13,14]. Animal models have shown that during aneurysm genesis, a combination of risk factors and hemodynamic stresses cause inflammatory responses in the endothelium and pro-inflammatory phenotypic changes in vascular smooth muscle cells [40,41,42]. These initial changes lead to the production of matrix metalloproteinases (MMPs) that degrade the extracellular matrix and lead to bulging of the artery [20,43]. The resultant aneurysm out-pouching is likely accompanied by local increase in chemokines and cytokines (IL-1β, IL-17, TNF-α) in blood of the aneurysm lumen [17]. These signals, in addition to a progressively leaky and sticky endothelial surface, likely attract circulating leukocytes that infiltrate the aneurysm tissue to further produce MMPs, degrading the IA wall and advancing its growth. This is evidenced by multiple histological analyses that have found leukocytes (mainly macrophages and T cells) in the walls of IAs [24,44], and gene expression studies that have reported increased matrix degradation, inflammatory processes, and cytokine/chemoattractant signaling [7,8,9,45,46,47,48].

The role of mononuclear cells has been widely studied in IA [49]. PBMCs arise from hematopoietic stem cells in the bone marrow via hematopoiesis, and are composed of myeloid cells (primarily monocytes), lymphoid cells (primarily T cells, B cells and NK cells), and dendritic cells. They encompass key components of both the innate and adaptive immune systems, which defend the body against infection and aid in the repair of diseased tissue. Of all the PBMCs, infiltrating monocytes (mainly M1 macrophages) have been the most widely studied in IA [23,50,51]. They have been shown to play significant roles in the degradation of the internal elastic lamina via NF-kB-mediated expression of MMP-2 and MMP-9 [52]. Macrophage expression of NF-kB also leads to upregulation of MCP-1, VCAM1, and IL-1B, which further contribute to the complex environment of inflammatory signaling and immune cell extravasation [49]. While less studied, both T and B lymphocytes are also present in IAs and can play a role in IA pathophysiology. In particular, studies have shown that, as with macrophages, T lymphocytes express pro-inflammatory cytokines, such as TNF-α, IFN-γ, and IL-6, that contribute to aneurysmal inflammation [24,53].

Considering the critical role of circulating PBMCs in IA inflammation and that they are actively recruited to the aneurysm wall from the blood, we hypothesized that expression patterns in PBMCs may be altered in patients harboring IAs compared to their IA-free counterparts. Transcriptome profiling by next-generation RNA sequencing identified a signature of 54 differentially expressed genes with an absolute fold change of ≥1.3 (40 increased, 14 decreased). The majority of these genes maintained the same expression pattern in an independent validation cohort of patients and could delineate patients with IA from controls based on our PCA and machine learning analysis (Ensemble classifier). To our knowledge, only one other study has investigated circulating gene expression patterns of IA in PBMCs. Sabatino et al. collected PBMCs from a cohort of 15 unruptured IAs, 15 ruptured IAs, and 15 control patients and performed gene-expression oligo-microarrays on isolated PBMC RNA [28]. In all, they identified 53 differentially expressed mRNAs between the unruptured IA and control groups (16 increased, 37 decreased) [28]. These upregulated genes were largely related to apoptosis and intracellular signaling, while the decreased genes were dominated by heat shock protein genes and genes related to the cytoskeleton and signal transduction [28]. While this study showed feasibility that IA was associated with differential expression of PBMCs, the results are difficult to interpret because: (A) they only analyzed a small dataset (implementing simple t-test to select differentially expressed genes), (B) they did not control their cohorts based on potentially confounding comorbidities/demographics, (C) they did not incorporate any validation into their analysis pipeline, and (D) they used microarray technology that is less sensitive and comprehensive than modern RNA sequencing techniques.

We designed the current study to avoid previous pitfalls and ensure greater accuracy of results. To do this, we confirmed diagnoses on DSA imaging, cohort-controlled our datasets, and implemented a small replication study. These measures helped to increase the likelihood that the discovered signature is truly associated with the presence of unruptured IA. Additionally, we used next-generation RNA sequencing. Unlike microarrays used in past studies, RNA sequencing offers a larger dynamic range, facilitating detection of expression differences in low-abundance transcripts, and avoids predetermined probes, allowing for examination of novel RNAs (i.e., splice variants and gene isoforms) [54,55]. Based on this experimental setup, we were able to discover an IA-associated signature of 54 genes. Similar to Sabatino et al., we found differential expression of heat shock proteins (*HSPA2*), as well as differential expression of many genes involved in intracellular signaling, such as g-protein coupled receptors (i.e., *OR1AK2*, *OMG*, *SSTR3*), and plasma membrane ion channels (i.e., *TRPV4*, *KCNG1*). Other IA-associated genes we identified have also been found to be differentially expressed in various blood components of IA patients. *ANKRD22*, which was increased in our study, was also increased in whole blood expression profiles as reported by Zhao et al. [56]. Expression differences in *CCR8*, *G0S2*, *SDC3* (all increased) and *UTS2* (decreased) were also seen in previous work from our group, in which we investigated expression signatures in circulating neutrophils as well as whole blood from patients with IA [6,7,9].

In this study, we detected many other significant genes, which broadly indicated peripheral activation of PBMCs during IA. Our bioinformatics analyses show that PBMCs from IA patients reflect a complex reaction to intravascular perturbations that is associated with increased cell activation, cell signaling, adhesion, and regulation of extracellular matrix. The activation of circulating immune cells, likely monocytes and T cells, is most reflected in enriched “response to stimulus” and “cell adhesion” ontologies. These are related to increased expression of *HLA-DQB2* (a major histocompatibility complex) and *CCR8*. *CCR8* is related to inflammatory signaling, as it is in the beta chemokine receptor family and has increased expression in M1 pro-inflammatory macrophages (compared to M2 macrophages), which have also been shown to be more prevalent in IAs and may contribute more to pathologic remodeling during IA [50,51]. Activation of PBMCs was also evident by the upregulation of the cell cycle gene, *G0S2* [57]. As demonstrated in our network analysis, this may occur through indirect interaction with NF-kB, a major node in Network 1, and a key player in inflammatory signaling of monocytes during IA. Furthermore, increased expression of *TIMD4* in IA PBMCs also indicates the activation of peripheral blood monocytes and T cells, as it has been a marker of recruited macrophages and macrophage polarization and is involved in T cell proliferation [58,59,60].

In addition to activation, gene ontology term enrichment analysis revealed increased structural development (evidenced by enrichment of the “anatomical structure morphogenesis” term). In our network analysis, this was conferred through several ADAMTS nodes and their connections to collagens and TGFB (a key inflammatory regulator in IA). Indeed *ADAMTS17*, a member of the ADAMTS family that is involved in proteoglycan cleavage along with MMPs [61,62], was increased in PBMCs from IA patients in our data. Another prominent enzymatic gene that was upregulated in our data is *PTGS2* or *COX2*. *COX2* is an inducible enzyme responsible for prostaglandin production, and as such, plays an essential role in inflammatory pathways [63,64]. Its expression has been associated with IA progression, as it is increased in ruptured IAs, larger IAs, and IAs with unstable walls [65,66]. One explanation for this relationship is that it has been observed to be increased in intraluminal thrombi, which line the IA wall and further trap circulating immune cells to create a hyper-degradative, cytotoxic environment [67,68,69]. We suspect that for PBMCs in IA, the regulation of *COX2*, and other important genes (i.e., *G0S2*, *TIMD4* and *CCR8*), is coordinated through TNF (in addition to NF-kB), as evidenced by our upstream analysis in IPA. TNFα is a critical part of the immune system and has been demonstrated to be a key mediator of inflammatory cell infiltrates in IA disease [25,70]. We suspect that TNF signaling may also be involved in priming of circulating PMBCs, namely monocytes, during IA.

Given the potential role of NF-kB and TNF (two key inflammatory mediators in IA tissue [71]) in regulating the observed expression differences in our study, we suspect an interaction between the PBMCs and the aneurysmal tissue (via contact or factors released into the blood). Therefore, we also sought to determine if any of the IA-associated genes were related to IA size and risk. Indeed, in past studies, we have demonstrated that differential expression in circulating neutrophils was exaggerated in patients with larger IAs. Here, correlation analysis showed significant positive relationships between *MKRN3* and *TRIM7*, and IA size and 5-year rupture risk (ISUIA). While the role of *MKRN3* is relatively unknown, *TRIM7* has been shown to promote TLR4-mediated signaling activation, which via NF-kB, leads to the production of proinflammatory cytokines and type I interferon [72]. This could indicate that greater inflammatory signaling occurs in peripheral immune cells of patients with larger or more unstable IAs. Conversely, we found significant negative relationships between *PHGDH* and *TIMD4*, and IA size and rupture risk. *PHGDH* encodes an enzyme that is involved in L-serine synthesis, and has been shown to have decreased expression in aneurysm tissue [73]. The negative relationship with IAs of greater size may support these findings in the IA tissue. Moreover, given the role of *TIMD4*, the correlation analysis may also indicate decreased T cell proliferation in patients with larger IAs. This may be akin to findings by Korostynski et al., who found a decrease in CD4+ lymphocytes in the blood of patients with ruptured IAs [74]. Larger, longitudinal studies will be needed to validate these findings and identify other circulating expression correlates of IA risk and instability.

In summary, transcriptome profiling of PBMCs in patients with IA revealed a signature of 54 significantly differentially expressed genes. These genes are likely to be related to biological processes known to be enriched in IAs, such as NF-kB signaling and TNF activity. Because of this relationship to the disease, we posit that this signature, upon future rigorous validation, may be a potential biomarker for IA presence. Previous work in this field has identified circulating protein markers, such as VEGF [75] or MCP-1 [70], as potential IA biomarkers. Yet, the ubiquity in the expression of such proteins across multiple other vascular diseases [76,77] limits their diagnostic potential. On the other hand, clinical studies have also shown that in IA, particularly after rupture, there is a change in cell populations (which was not observed in our data); neutrophil-to-lymphocyte ratios increase [18], while lymphocyte-to-monocyte ratios decrease [74], suggesting increases in neutrophils and monocytes. However, these ratios may be best suited for IA prognosis, as the ratio of immune cells is often altered in many other disease states [78,79]. More recent studies by our group [6,7,8,9,80] and others [28,56,81,82] have shown significant differences in panels of genes from circulating inflammatory cells, such as neutrophils, in patients with IA, which could be more specific to the disease. Indeed, our preliminary machine learning analysis highlights this exciting potential for our PBMC RNA signature, although rigorous validations are critically required.

### Limitations

This preliminary study has several limitations. First, our study had a relatively small sample size. However, demonstrating separation of IAs from controls in an independent cohort gives confidence in the identified signature. Future studies in larger cohorts will increase the statistical power in identifying differentially expressed genes. Second, the study subjects were recruited from patients receiving cerebral imaging (which was necessary to confirm the presence or absence of IA) at a single center. This may have introduced a potential selection bias. Future studies in broader, randomized populations from multiple centers are needed. Third, despite careful patient selection to ensure matched cohorts, there were some differences (albeit not significant differences) in the rates of patients’ characteristics, such as smoking and osteoarthritis, which could affect the data. Future efforts in larger datasets should be made to normalize results based on patient demographics and comorbidities. Lastly, there is a possibility that the expression signature could be influenced by other conditions not accounted for. Additional studies should investigate the specificity of the differential expression profiles to IA.

## 5. Conclusions

In this preliminary study, we performed transcriptome profiling on PBMCs from patients with and without IAs and identified a signature of 54 differentially expressed genes with an absolute fold-change of ≥1.3 and *q*-value < 0.05. These genes also separated patients with IAs from controls in a small validation cohort. Bioinformatics analyses demonstrated enrichment of structural regulation processes, intracellular signaling function and regulation of ion transport. IPA analysis showed that these processes were likely coordinated through NF-kB, cytokine signaling, growth factors, and TNF activity. Our machine learning analysis highlights the exciting potential to develop circulating RNA expression-based diagnostics for IA in future, larger studies.

## Figures and Tables

**Table 1:** Patient characteristics of the discovery cohort *.

	Patients with IA (*n* = 18)	Patients without IA (*n* = 21)	*p*-Value
**Age (years)** (mean + SE)	56.5 ± 2.86	56.5 ± 3.31	0.996
[Q2 (Q1/Q3)]	55.5 (50/62.75)	50 (47/68)	
**Sex**			
Female	66.67%	71.43%	1
**Current Smoker**			
Yes	27.78%	4.76%	0.0775
**Comorbidities**			
Osteoarthritis	0.00%	14.29%	0.235
Diabetes mellitus	0.00%	19.05%	0.11
Heart disease	16.67%	19.05%	1
Hyperlipidemia	22.22%	38.10%	0.322
Hypertension	33.33%	38.10%	1
Stroke history	0.00%	4.76%	1

* Clinical characteristics of the randomly selected discovery cohort. These clinical factors were retrieved from patients’ medical records. With the exception of age, these data points were quantified as binary data points. Significant differences between each group were evaluated with a Student’s *t*-test for continuous data (age) and a Fisher’s exact test for categorical data (α = 0.05). There was no statistically significant difference in age, sex, smoking, or comorbidities between the IA and control groups. (Abbreviations: IA = intracranial aneurysm, *n* = number, Q = quartile, SE = standard error).

**Table 2:** Differentially expressed genes identified in the discovery cohort *.

Gene	Ensembl ID	Log2(F-C)	*q*-Value
*ANKRD24*	ENSG00000089847.12	1.02440061	1.7433 × 10^−13^
*HLA-DQB2*	ENSG00000232629.8	0.96482544	0.04366232
*OR2AK2*	ENSG00000187080.7	0.74946987	0.02309426
*PHOSPHO1*	ENSG00000173868.11	0.70543487	0.03393937
*ANKRD22*	ENSG00000152766.5	0.69650906	0.00201465
*SLC7A8*	ENSG00000092068.18	0.65335667	0.03981758
*COCH*	ENSG00000100473.15	0.64742593	0.0453735
*SORCS3*	ENSG00000156395.12	0.63403617	0.01399435
*CCR8*	ENSG00000179934.6	0.5985602	0.01680311
*EGR2*	ENSG00000122877.13	0.5965057	0.01284844
*G0S2*	ENSG00000123689.5	0.57278717	0.00814323
*ZNF135*	ENSG00000176293.19	0.5427691	0.00793924
*OMG*	ENSG00000126861.4	0.52775651	0.02057129
*CNTNAP1*	ENSG00000108797.11	0.51886293	0.04669498
*PTGS2*	ENSG00000073756.11	0.50884249	0.01626401
*ADAMTS17*	ENSG00000140470.13	0.50648661	1.664 × 10^−6^
*FAM83D*	ENSG00000101447.13	0.50468148	0.04240973
*SYNPO*	ENSG00000171992.12	0.49739469	0.04915655
*SMN1*	ENSG00000172062.16	0.48613932	1.9529 × 10^−5^
*MKRN3*	ENSG00000179455.7	0.47839381	0.02970793
*TRPV4*	ENSG00000111199.10	0.47241322	0.00038868
*KCNG1*	ENSG00000026559.13	0.45359347	0.02898435
*SDC3*	ENSG00000162512.15	0.43993667	0.00201465
*MEOX1*	ENSG00000005102.12	0.43878267	0.04189198
*FAM71F2*	ENSG00000205085.8	0.43702203	0.03651116
*TIMD4*	ENSG00000145850.8	0.43318811	0.02661721
*TRIM7*	ENSG00000146054.17	0.43251954	0.01502045
*ODF3B*	ENSG00000177989.13	0.42500275	0.02928318
*SHISA8*	ENSG00000234965.2	0.42403186	0.02784039
*HSPA2*	ENSG00000126803.9	0.41906929	0.04206239
*C7orf61*	ENSG00000185955.4	0.41889115	0.03017211
*CXorf67*	ENSG00000187690.3	0.41696671	0.02238062
*ANO5*	ENSG00000171714.10	0.40898144	0.02784039
*FMN1*	ENSG00000248905.8	0.40803563	0.02269361
*RP11-231C14.4*	ENSG00000169203.16	0.40751277	0.04859605
*RAVER2*	ENSG00000162437.14	0.39236484	1.1939 × 10^−8^
*ANKRD34B*	ENSG00000189127.7	0.39138387	0.02561036
*SCARF2*	ENSG00000244486.7	0.3850027	0.02164902
*CACNA1I*	ENSG00000100346.17	0.38424407	0.02057129
*SSTR3*	ENSG00000278195.1	0.38405987	0.00524454
*SAMD14*	ENSG00000167100.14	−0.4203139	0.02550125
*TRNP1*	ENSG00000253368.3	−0.4552427	0.04311265
*CDCP1*	ENSG00000163814.7	−0.4854855	0.00524454
*FOLR3*	ENSG00000110203.8	−0.4935978	0.01029719
*PHGDH*	ENSG00000092621.11	−0.5333702	0.02265579
*PDZK1IP1*	ENSG00000162366.7	−0.5388676	0.04025004
*BOK*	ENSG00000176720.4	−0.5554993	0.01106655
*RETN*	ENSG00000104918.7	−0.5886811	0.01718698
*DEFA4*	ENSG00000164821.4	−0.6427006	0.03699697
*KIR3DL2*	ENSG00000240403.5	−0.6593919	0.04000571
*TNNT1*	ENSG00000105048.16	−0.771146	0.00964492
*L1TD1*	ENSG00000240563.1	−0.834892	0.03447339
*PRTN3*	ENSG00000196415.9	−0.9982895	0.02189969
*UTS2*	ENSG00000049247.13	−1.2567442	1.4788 × 10^−7^

* Significantly differentially expressed transcripts with *q*-value < 0.05 and an absolute fold-change ≥ 1.3. 40 genes had higher expression in IA, and 14 genes had lower expression in IA. (Abbreviations: F-C = fold-change).

## Data Availability

The data presented in this study are available on request from the corresponding author. The data are not publicly available due to continued analysis by the corresponding author’s research team and ongoing IP considerations.

## Supplementary Materials

The following are available online at https://www.mdpi.com/article/10.3390/diagnostics11061092/s1, Table S1: Aneurysm Characteristics of the Discovery Cohort, Table S2: RNA Quality, Table S3: Sequencing Quality, Table S4: Significantly Enriched Gene Ontologies, Table S5: Additional Information on Significant Networks from IPA, Table S6: Additional Information on Upstream Regulators from IPA, Table S7: Patient Characteristics of the Validation Cohort, Table S8: Aneurysm Characteristics of the Validation Cohort, Table S9: All Correlation Analysis Results, Figure S1: Distribution of cell types in isolated PBMCs of both groups computed by CIBERSORT, Figure S2: Comparison in fold-change of differentially expressed gene in the discovery cohort compared to the validation cohort.
