# Supplementary material for: Identification of Circulating Gene Expression Signatures of Intracranial Aneurysm in Peripheral Blood Mononuclear Cells
Source: Diagnostics (Basel). 2021 Jun 15;11(6):1092. doi: 10.3390/diagnostics11061092 (PMC8232768; doi:10.3390/diagnostics11061092)
Supplement: Supplementary file 1 [file diagnostics-11-01092-s001.zip › diagnostics-1244600-supplementary.pdf]

Supplemental Information for: Identification of Circulating Gene Expression Signatures of Intracranial Aneurysm in Peripheral Blood Mononuclear Cells

*Vincent M. Tutino PhD[1,2,3,4], Haley R. Zebraski MS[5], Hamidreza Rajabzadeh-Oghaz PhD[1,3], Muhammad Waqas MBBS[1,3], James N. Jarvis MD[6], Konrad Bach MD[7], Maxim Mokin MD-PhD[7], Kenneth V. Snyder MD-PhD[1,3], Adnan H. Siddiqui MD-PhD[1,3], Kerry E. Poppenberg PhD[1,3]

[1]Canon Stroke and Vascular Research Center; [2]Department of Pathology and Anatomical Sciences, [3]Department of Neurosurgery, [4]Department of Mechanical and Aerospace Engineering, [5]Department of Biomedical Engineering, [6]Department of Pediatrics University at Buffalo, Buffalo, NY, USA; [7]Department of Neurosurgery and Brain Repair, University of South Florida, Tampa, FL, USA

***Corresponding Author:**
Vincent M. Tutino, PhD
Canon Stroke and Vascular Research Center
Clinical Translational Research Center
875 Ellicott Street
Buffalo, New York 14203 USA
Email: vincentt@buffalo.edu
Phone: (716) 829-5400
Fax: (716) 854-1850

Supplemental Tables

Table S1: Aneurysm Characteristics of the Discovery Cohort.*

Characteristic	Value
**Family history of IA (n/n_{total})**	3/18 (16.7%)
**Patients with Multiple IAs (n/n_{total})**	1/18 (5.6%)
**Size (mm) [mean (min/max)]**	6.2 (2/18)
**IA Location (n/n_{total})**	
ACom	1/19 (5.3%)
BA	1/19 (5.3%)
Cavernous ICA	2/19 (10.5%)
ICA	6/19 (31.6%)
MCA	5/19 (26.3%)
PCom/PCA	4/19 (21.1%)

*Aneurysm size ranged from 2 mm to 18 mm. The aneurysms were situated at various locations in the Circle of Willis, with most being around the internal carotid artery and its branches. Three patients with IAs had a family history of the disease. (Abbreviations: ACom=anterior communicating artery, BA=basilar Artery, IA=intracranial aneurysm, ICA=internal carotid artery, MCA=middle cerebral artery, n=number, PCA=posterior cerebellar artery, PCom=posterior communicating artery)

Table S2: RNA Quality.*

ID	Class	260/280	RQN
Control 1	Discovery	2.07	8.7
Control 2	Discovery	2.06	8.0
Control 3	Discovery	2.09	9.9
Control 4	Discovery	2.07	9.6
Control 5	Discovery	2.09	8.5
Control 6	Discovery	2.11	9.2
Control 7	Discovery	2.09	10.0
Control 8	Discovery	2.05	10.0
Control 9	Discovery	1.97	10.0
Control 10	Discovery	2.07	10.0
Control 11	Discovery	2.08	10.0
Control 12	Discovery	2.09	10.0
Control 13	Discovery	2.13	9.2
Control 14	Discovery	2.07	9.9
Control 15	Discovery	2.10	9.3
Control 16	Discovery	2.10	8.4
Control 17	Discovery	2.10	9.1
Control 18	Discovery	2.11	9.3
Control 19	Discovery	2.09	9.2
Control 20	Discovery	2.11	10.0
Control 21	Discovery	2.07	9.3
Control 22	Validation	2.06	9.5
Control 23	Validation	2.09	9.3
Control 24	Validation	2.05	9.6
Control 25	Validation	2.08	9.7
Control 26	Validation	2.05	9.6
Control 27	Validation	2.05	9.2
Control 28	Validation	2.07	9.1
Aneurysm 1	Discovery	2.09	8.8
Aneurysm 2	Discovery	2.08	9.7
Aneurysm 3	Discovery	2.08	9.9
Aneurysm 4	Discovery	2.08	8.9
Aneurysm 5	Discovery	2.04	9.7
Aneurysm 6	Discovery	2.09	8.1
Aneurysm 7	Discovery	2.06	8.9
Aneurysm 8	Discovery	2.07	8.2
Aneurysm 9	Discovery	2.01	9.7
Aneurysm 10	Discovery	2.06	9.0
Aneurysm 11	Discovery	2.08	9.7
Aneurysm 12	Discovery	2.10	9.7
Aneurysm 13	Discovery	2.09	9.8
Aneurysm 14	Discovery	2.05	9.5
Aneurysm 15	Discovery	2.09	9.6
Aneurysm 16	Discovery	2.09	10.0
Aneurysm 17	Discovery	2.08	9.9
Aneurysm 18	Discovery	2.07	8.7
Aneurysm 19	Validation	2.08	10.0
Aneurysm 20	Validation	2.06	10.0

Aneurysm 21	Validation	2.06	9.4
Aneurysm 22	Validation	2.94	8.7
Aneurysm 23	Validation	2.09	9.9
Aneurysm 24	Validation	2.08	9.6

* The quality of the RNA samples was assessed by the 260/280 ratio and the RQN. (Abbreviations: RQN=RNA quality number)

Table S3: Sequencing Quality.*

ID	Class	M Seqs	% Aligned
Control 1	Discovery	25.7	95.7%
Control 2	Discovery	33.7	92.3%
Control 3	Discovery	25.7	96.2%
Control 4	Discovery	30.1	95.7%
Control 5	Discovery	26.4	96.8%
Control 6	Discovery	26.6	96.6%
Control 7	Discovery	43.2	96.8%
Control 8	Discovery	43.6	97.0%
Control 9	Discovery	38.7	96.9%
Control 10	Discovery	51.3	97.1%
Control 11	Discovery	32.1	96.4%
Control 12	Discovery	29.1	96.3%
Control 13	Discovery	30.4	96.2%
Control 14	Discovery	27.2	97.0%
Control 15	Discovery	29.9	96.7%
Control 16	Discovery	25.2	97.1%
Control 17	Discovery	26.0	97.1%
Control 18	Discovery	26.7	97.0%
Control 19	Discovery	33.8	94.2%
Control 20	Discovery	30.4	97.3%
Control 21	Discovery	24.4	96.8%
Control 22	Validation	39.4	95.7%
Control 23	Validation	34.2	94.6%
Control 24	Validation	44.4	95.6%
Control 25	Validation	34.1	93.6%
Control 26	Validation	37.3	96.5%
Control 27	Validation	26.5	96.5%
Control 28	Validation	30.0	96.2%
Aneurysm 1	Discovery	36.2	95.3%
Aneurysm 2	Discovery	28.9	96.3%
Aneurysm 3	Discovery	27.5	96.4%
Aneurysm 4	Discovery	28.9	94.7%
Aneurysm 5	Discovery	31.6	97.2%
Aneurysm 6	Discovery	28.6	96.9%
Aneurysm 7	Discovery	36.8	96.9%
Aneurysm 8	Discovery	41.3	96.9%
Aneurysm 9	Discovery	29.7	97.2%
Aneurysm 10	Discovery	33.9	97.0%
Aneurysm 11	Discovery	30.7	96.5%
Aneurysm 12	Discovery	28.8	97.0%
Aneurysm 13	Discovery	27.1	96.8%
Aneurysm 14	Discovery	32.6	97.2%
Aneurysm 15	Discovery	23.4	95.1%
Aneurysm 16	Discovery	25.1	95.9%
Aneurysm 17	Discovery	32.1	95.6%
Aneurysm 18	Discovery	29.9	96.9%
Aneurysm 19	Validation	37.8	96.2%
Aneurysm 20	Validation	29.3	95.8%

Aneurysm 21	Validation	36.2	97.1%
Aneurysm 22	Validation	36.6	97.2%
Aneurysm 23	Validation	35.5	96.9%
Aneurysm 24	Validation	38.9	97.0%

* The quality of the RNA sequencing experiments was measure post-alignment via MultiQC. The report showed that the sequencing experiments had an average of 32.2 million mapped reads with a 96.3% aligned rate. (Abbreviations: M=million, Seqs=sequences)

Table S4: Significantly Enriched Gene Ontologies.*

Category	GO ID	GO Name	q-value	Focus Molecules
Genes with Increased Expression in IA				
Biological Process	GO:0008150	biological process	2.47E-07	SSTR3, CACNA1I, SCARF2, RAVER2, FMN1, ANO5, HSPA2, SHISA8, TRIM7, MEOX1, SDC3, KCNG1, TRPV4, MKRN3, SYNPO, FAM83D, ADAMTS17, PTGS2, CNTNAP1, OMG, ZNF135, G0S2, EGR2, CCR8, SORCS3, COCH, SLC7A8, PHOSPHO1, OR2AK2, HLA-DQB2
Biological Process	GO:0009987	cellular process	5.85E-07	SSTR3, CACNA1I, SCARF2, RAVER2, FMN1, ANO5, HSPA2, SHISA8, TRIM7, MEOX1, SDC3, KCNG1, TRPV4, MKRN3, SYNPO, FAM83D, ADAMTS17, PTGS2, CNTNAP1, OMG, ZNF135, G0S2, EGR2, CCR8, SORCS3, SLC7A8, PHOSPHO1, OR2AK2, HLA-DQB2
Biological Process	GO:0065007	biological regulation	0.000116	SSTR3, CACNA1I, FMN1, HSPA2, SHISA8, MEOX1, KCNG1, TRPV4, SYNPO, FAM83D, PTGS2, CNTNAP1, OMG, ZNF135, G0S2, EGR2, CCR8, SORCS3, COCH, SLC7A8, PHOSPHO1, OR2AK2, HLA-DQB2
Biological Process	GO:0050789	regulation of biological process	0.000235	SSTR3, CACNA1I, FMN1, HSPA2, SHISA8, MEOX1, KCNG1, TRPV4, SYNPO, FAM83D, PTGS2, CNTNAP1, OMG, ZNF135, G0S2, EGR2, CCR8, SORCS3, COCH, PHOSPHO1, OR2AK2, HLA-DQB2
Biological Process	GO:0032501	multicellular organismal process	0.000282	SSTR3, CACNA1I, FMN1, HSPA2, MEOX1, TRPV4, FAM83D, PTGS2, CNTNAP1, OMG, G0S2, EGR2, SORCS3, COCH, SLC7A8, PHOSPHO1, OR2AK2, HLA-DQB2
Biological Process	GO:0050794	regulation of cellular process	0.002889	SSTR3, CACNA1I, FMN1, HSPA2, SHISA8, MEOX1, KCNG1, TRPV4, SYNPO, FAM83D, PTGS2, CNTNAP1,

				OMG, ZNF135, G0S2, EGR2, CCR8, SORCS3, OR2AK2, HLA-DQB2
Biological Process	GO:0065008	regulation of biological quality	0.005548	CACNA1I, FMN1, SHISA8, TRPV4, PTGS2, CNTNAP1, G0S2, EGR2, CCR8, SORCS3, COCH, SLC7A8
Biological Process	GO:0009653	anatomical structure morphogenesis	0.007229	FMN1, MEOX1, TRPV4, PTGS2, CNTNAP1, OMG, ZNF135, EGR2, COCH, PHOSPHO1
Biological Process	GO:0048856	anatomical structure development	0.010642	SSTR3, FMN1, HSPA2, MEOX1, TRPV4, FAM83D, PTGS2, CNTNAP1, OMG, ZNF135, EGR2, COCH, PHOSPHO1, HLA-DQB2
Biological Process	GO:0006812	cation transport	0.010708	CACNA1I, HSPA2, SHISA8, KCNG1, TRPV4, PTGS2, SLC7A8
Biological Process	GO:0003008	system process	0.011273	CACNA1I, TRPV4, PTGS2, CNTNAP1, EGR2, SORCS3, COCH, SLC7A8, OR2AK2
Biological Process	GO:0050896	response to stimulus	0.01803	SSTR3, CACNA1I, HSPA2, SHISA8, TRPV4, FAM83D, PTGS2, CNTNAP1, OMG, G0S2, EGR2, CCR8, SORCS3, COCH, SLC7A8, OR2AK2, HLA-DQB2
Biological Process	GO:0098655	cation transmembrane transport	0.027353	CACNA1I, HSPA2, SHISA8, KCNG1, TRPV4, SLC7A8
Biological Process	GO:0032502	developmental process	0.027443	SSTR3, FMN1, HSPA2, MEOX1, TRPV4, FAM83D, PTGS2, CNTNAP1, OMG, ZNF135, EGR2, COCH, PHOSPHO1, HLA-DQB2
Biological Process	GO:0034220	ion transmembrane transport	0.029008	CACNA1I, ANO5, HSPA2, SHISA8, KCNG1, TRPV4, SLC7A8
Biological Process	GO:0050877	nervous system process	0.033083	CACNA1I, PTGS2, CNTNAP1, EGR2, SORCS3, COCH, OR2AK2
Biological Process	GO:0051716	cellular response to stimulus	0.036248	SSTR3, CACNA1I, HSPA2, SHISA8, TRPV4, FAM83D, PTGS2, CNTNAP1, OMG, G0S2, EGR2, CCR8, SORCS3, OR2AK2, HLA-DQB2
Biological Process	GO:0040011	locomotion	0.038102	CACNA1I, SDC3, TRPV4, FAM83D, PTGS2, EGR2, CCR8, SLC7A8

Biological Process	GO:0051179	localization	0.043439	CACNA1I, SCARF2, ANO5, HSPA2, SHISA8, SDC3, KCNG1, TRPV4, FAM83D, PTGS2, CNTNAP1, EGR2, CCR8, SLC7A8
Biological Process	GO:0007155	cell adhesion	0.044145	SCARF2, FMN1, TRPV4, CNTNAP1, OMG, CCR8, HLA-DQB2
Biological Process	GO:0022610	biological adhesion	0.045479	SCARF2, FMN1, TRPV4, CNTNAP1, OMG, CCR8, HLA-DQB2
Cellular Component	GO:0005575	cellular component	2.31E-12	SSTR3, CACNA1I, SCARF2, ANKRD34B, RAVER2, FMN1, ANO5, C7ORF61, HSPA2, SHISA8, ODF3B, TRIM7, TIMD4, MEOX1, SDC3, KCNG1, TRPV4, MKRN3, SYNPO, FAM83D, ADAMTS17, PTGS2, CNTNAP1, OMG, ZNF135, G0S2, EGR2, CCR8, SORCS3, COCH, SLC7A8, PHOSPHO1, OR2AK2, HLA-DQB2
Cellular Component	GO:0110165	cellular anatomical entity	3.42E-11	SSTR3, CACNA1I, SCARF2, ANKRD34B, RAVER2, FMN1, ANO5, C7ORF61, HSPA2, SHISA8, ODF3B, TRIM7, TIMD4, MEOX1, SDC3, KCNG1, TRPV4, SYNPO, FAM83D, ADAMTS17, PTGS2, CNTNAP1, OMG, ZNF135, G0S2, EGR2, CCR8, SORCS3, COCH, SLC7A8, PHOSPHO1, OR2AK2, HLA-DQB2
Cellular Component	GO:0071944	cell periphery	5.09E-09	SSTR3, CACNA1I, FMN1, ANO5, HSPA2, SHISA8, SDC3, KCNG1, TRPV4, ADAMTS17, PTGS2, CNTNAP1, OMG, CCR8, SORCS3, COCH, SLC7A8, PHOSPHO1, OR2AK2, HLA-DQB2
Cellular Component	GO:0005886	plasma membrane	1.06E-06	SSTR3, CACNA1I, FMN1, ANO5, HSPA2, SHISA8, SDC3, KCNG1, TRPV4, PTGS2, CNTNAP1, OMG, CCR8, SORCS3, SLC7A8, OR2AK2, HLA-DQB2
Cellular Component	GO:0031224	intrinsic component of membrane	1.73E-06	SSTR3, CACNA1I, SCARF2, ANO5, HSPA2, SHISA8, TIMD4, SDC3, KCNG1, TRPV4, CNTNAP1, OMG, CCR8,

				SORCS3, SLC7A8, OR2AK2, HLA-DQB2
Cellular Component	GO:0016021	integral component of membrane	9.1E-06	SSTR3, CACNA1I, SCARF2, ANO5, HSPA2, SHISA8, TIMD4, SDC3, KCNG1, TRPV4, CNTNAP1, CCR8, SORCS3, SLC7A8, OR2AK2, HLA-DQB2
Cellular Component	GO:0005887	integral component of plasma membrane	6.35E-05	SSTR3, CACNA1I, SHISA8, KCNG1, TRPV4, CNTNAP1, CCR8, SORCS3, SLC7A8
Cellular Component	GO:0031226	intrinsic component of plasma membrane	9.46E-05	SSTR3, CACNA1I, SHISA8, KCNG1, TRPV4, CNTNAP1, CCR8, SORCS3, SLC7A8
Cellular Component	GO:0016020	membrane	0.000112	SSTR3, CACNA1I, SCARF2, FMN1, ANO5, HSPA2, SHISA8, TIMD4, SDC3, KCNG1, TRPV4, PTGS2, CNTNAP1, OMG, CCR8, SORCS3, SLC7A8, OR2AK2, HLA-DQB2
Cellular Component	GO:0043226	organelle	0.000292	SSTR3, ANKRD34B, RAVER2, FMN1, ANO5, C7ORF61, HSPA2, SHISA8, ODF3B, TRIM7, MEOX1, SDC3, TRPV4, SYNPO, FAM83D, PTGS2, ZNF135, G0S2, EGR2, SORCS3, PHOSPHO1, HLA-DQB2
Cellular Component	GO:0120025	plasma membrane bounded cell projection	0.000767	SSTR3, CACNA1I, SHISA8, SDC3, TRPV4, SYNPO, PTGS2, CNTNAP1, SLC7A8
Cellular Component	GO:0042995	cell projection	0.001096	SSTR3, CACNA1I, SHISA8, SDC3, TRPV4, SYNPO, PTGS2, CNTNAP1, SLC7A8
Cellular Component	GO:0098590	plasma membrane region	0.001151	SSTR3, SHISA8, TRPV4, PTGS2, CNTNAP1, SORCS3, SLC7A8
Cellular Component	GO:0043005	neuron projection	0.002293	SSTR3, CACNA1I, SHISA8, TRPV4, SYNPO, PTGS2, CNTNAP1
Cellular Component	GO:0005622	intracellular anatomical structure	0.003344	SSTR3, ANKRD34B, RAVER2, FMN1, ANO5, C7ORF61, HSPA2, ODF3B, TRIM7, MEOX1, SDC3, TRPV4, SYNPO, FAM83D, PTGS2, ZNF135, G0S2, EGR2, SLC7A8, PHOSPHO1, HLA-DQB2

Cellular Component	GO:0098797	plasma membrane protein complex	0.008091	CACNA1I, HSPA2, SHISA8, KCNG1, HLA-DQB2
Cellular Component	GO:0005737	cytoplasm	0.008933	SSTR3, ANKRD34B, RAVER2, FMN1, ANO5, HSPA2, TRIM7, MEOX1, SDC3, TRPV4, SYNPO, FAM83D, PTGS2, G0S2, EGR2, SLC7A8, PHOSPHO1, HLA-DQB2
Cellular Component	GO:0043229	intracellular organelle	0.017065	ANKRD34B, RAVER2, FMN1, ANO5, C7ORF61, HSPA2, ODF3B, TRIM7, MEOX1, SDC3, TRPV4, SYNPO, FAM83D, PTGS2, ZNF135, G0S2, EGR2, HLA-DQB2
Cellular Component	GO:0070161	anchoring junction	0.019326	SCARF2, FMN1, TRPV4, SYNPO, CNTNAP1
Cellular Component	GO:0043227	membrane-bounded organelle	0.025159	SSTR3, ANKRD34B, RAVER2, FMN1, ANO5, C7ORF61, HSPA2, TRIM7, MEOX1, SDC3, TRPV4, SYNPO, PTGS2, ZNF135, G0S2, EGR2, PHOSPHO1, HLA-DQB2
Cellular Component	GO:0009986	cell surface	0.027053	HSPA2, SDC3, TRPV4, CCR8, HLA-DQB2
Cellular Component	GO:0030054	cell junction	0.032222	SCARF2, FMN1, SHISA8, TRPV4, SYNPO, CNTNAP1, SORCS3
Molecular Function	GO:0003674	molecular function	2.33E-12	SSTR3, CACNA1I, SCARF2, ANKRD34B, RAVER2, FMN1, ANO5, HSPA2, ODF3B, TRIM7, FAM71F2, MEOX1, SDC3, KCNG1, TRPV4, MKRN3, SYNPO, FAM83D, ADAMTS17, PTGS2, CNTNAP1, OMG, ZNF135, G0S2, EGR2, CCR8, SORCS3, COCH, SLC7A8, ANKRD22, PHOSPHO1, OR2AK2, HLA-DQB2, ANKRD24
Molecular Function	GO:0005488	binding	2.7E-12	SSTR3, CACNA1I, SCARF2, ANKRD34B, RAVER2, FMN1, ANO5, HSPA2, ODF3B, TRIM7, FAM71F2, MEOX1, SDC3, KCNG1, TRPV4, MKRN3, SYNPO, FAM83D, ADAMTS17, PTGS2, CNTNAP1, OMG, ZNF135, G0S2, EGR2, CCR8, SORCS3, COCH, SLC7A8, ANKRD22, PHOSPHO1, HLA-DQB2, ANKRD24

Molecular Function	GO:0005515	protein binding	9.5E-12	SSTR3, CACNA1I, SCARF2, ANKRD34B, FMN1, ANO5, HSPA2, ODF3B, TRIM7, FAM71F2, MEOX1, SDC3, KCNG1, TRPV4, MKRN3, SYNPO, FAM83D, PTGS2, CNTNAP1, OMG, ZNF135, G0S2, EGR2, CCR8, SORCS3, COCH, SLC7A8, ANKRD22, PHOSPHO1, HLA-DQB2, ANKRD24
Molecular Function	GO:0019904	protein domain specific binding	0.000927	FMN1, HSPA2, MEOX1, TRPV4, CNTNAP1, EGR2
Molecular Function	GO:0060089	molecular transducer activity	0.005399	SSTR3, TRPV4, CNTNAP1, CCR8, SORCS3, OR2AK2, HLA-DQB2
Molecular Function	GO:0038023	signaling receptor activity	0.005399	SSTR3, TRPV4, CNTNAP1, CCR8, SORCS3, OR2AK2, HLA-DQB2
Molecular Function	GO:0004888	transmembrane signaling receptor activity	0.02384	SSTR3, TRPV4, CCR8, SORCS3, OR2AK2, HLA-DQB2
Molecular Function	GO:0015318	inorganic molecular entity transmembrane transporter activity	0.031136	CACNA1I, ANO5, KCNG1, TRPV4, SLC7A8

Genes with Decreased Expression in IA

Biological Process	GO:0032501	multicellular organismal process	0.010904	UTS2, PRTN3, TNNT1, RETN, BOK, PHGDH, FOLR3, TRNP1, SAMD14
Biological Process	GO:0009987	cellular process	0.011832	UTS2, PRTN3, L1TD1, TNNT1, KIR3DL2, DEFA4, RETN, BOK, PHGDH, FOLR3, TRNP1, SAMD14
Biological Process	GO:0008150	biological process	0.02115	UTS2, PRTN3, L1TD1, TNNT1, KIR3DL2, DEFA4, RETN, BOK, PHGDH, FOLR3, TRNP1, SAMD14
Biological Process	GO:0050896	response to stimulus	0.044048	UTS2, PRTN3, TNNT1, KIR3DL2, DEFA4, RETN, BOK, FOLR3, SAMD14
Cellular Component	GO:0110165	cellular anatomical entity	1.66E-05	UTS2, PRTN3, L1TD1, TNNT1, KIR3DL2, DEFA4, RETN, BOK, PDZK1IP1, PHGDH, FOLR3, CDCP1, TRNP1, SAMD14
Cellular Component	GO:0005575	cellular component	1.85E-05	UTS2, PRTN3, L1TD1, TNNT1, KIR3DL2, DEFA4, RETN, BOK, PDZK1IP1, PHGDH, FOLR3, CDCP1, TRNP1, SAMD14

Cellular Component	GO:0005576	extracellular region	0.000444	UTS2, PRTN3, DEFA4, RETN, PDZK1IP1, PHGDH, FOLR3, CDCP1
Cellular Component	GO:0031982	vesicle	0.002914	PRTN3, DEFA4, RETN, BOK, PDZK1IP1, PHGDH, FOLR3
Cellular Component	GO:0043226	organelle	0.003874	PRTN3, L1TD1, TNNT1, DEFA4, RETN, BOK, PDZK1IP1, PHGDH, FOLR3, TRNP1, SAMD14
Cellular Component	GO:0005615	extracellular space	0.018592	UTS2, PRTN3, DEFA4, RETN, PDZK1IP1, PHGDH
Cellular Component	GO:0031410	cytoplasmic vesicle	0.032618	PRTN3, DEFA4, RETN, BOK, FOLR3
Cellular Component	GO:0097708	intracellular vesicle	0.032871	PRTN3, DEFA4, RETN, BOK, FOLR3
Molecular Function	GO:0005488	binding	4.28E-06	UTS2, PRTN3, L1TD1, TNNT1, KIR3DL2, DEFA4, RETN, BOK, PDZK1IP1, PHGDH, FOLR3, CDCP1, TRNP1, SAMD14
Molecular Function	GO:0003674	molecular function	1.5E-05	UTS2, PRTN3, L1TD1, TNNT1, KIR3DL2, DEFA4, RETN, BOK, PDZK1IP1, PHGDH, FOLR3, CDCP1, TRNP1, SAMD14
Molecular Function	GO:0005515	protein binding	0.000517	UTS2, PRTN3, L1TD1, TNNT1, KIR3DL2, DEFA4, RETN, BOK, PDZK1IP1, FOLR3, CDCP1, SAMD14
Molecular Function	GO:0005102	signaling receptor binding	0.005591	UTS2, PRTN3, KIR3DL2, RETN, BOK

* Significant ontologies with a q-value<0.05 and five or more focus molecules are reported.
(Abbreviations: GO=gene ontology)

Table S5: Additional Information on Significant Networks From IPA.*

Network	Molecules in Network	P-score	Focus Molecules	Top Diseases and Functions
1	26s Proteasome, Actin, BOK, CDCP1, CG, cytokine, EGR2, ERK, ERK1/2, FMN1, G0S2, Histone h3, Ige, IgG, Immunoglobulin, Insulin, LDL, Mek, NFkB (complex), P38 MAPK, Pka, PRKAA, PRTN3, PTGS2, RETN, RNA polymerase II, SCARF2, SDC3, SMN1/SMN2, SORCS3, SSTR3, TIMD4, TRPV4, Ubiquitin, UTS2	36	16	Behavior, Cell Death and Survival, Connective Tissue Disorders
2	ADAM19, ADAMTS17, ADAMTS4, AGR3, ANKRD22, ANO5, beta-glycerophosphoric acid, BOD1L1, CGB3 (includes others), COCH, COL11A1, COL1A1, Collagen type VII, CUL1, desoxycorticosterone, Ecm, EGFR, Endothelin, EZH2, HSPA2, L1TD1, LOC102724788/PRODH, MGAT5, MGP, mir-101, MYC, OMG, PDZK1IP1, PHGDH, SLC3A1, SLC7A8, TFRC, TGFB1, THSD4, ZFP42	20	10	Amino Acid Metabolism, Cell Cycle, Cellular Development
3	ACBD4, ANTKMT, APEX2, AR, CNTNAP1, DDX5, DNA-methyltransferase, EGR4, EI24, HDAC2, HEPH, HPGD, KCNG1, MEOX1, mir-143, MKRN3, MYOZ2, NABP1, ODF3B, OR2AK2, PEX26, PLEKHF1, RAVER2, RBM41, SHISA8, SMARCA4, SRF, TBPL1, TNNI2, TNNT1, TNNT2, TP53, TRNP1, Troponin t, TSHZ3	20	10	Cardiovascular System Development and Function, Cellular Assembly and Organization, Cellular Development

* Significant networks with a P-score ≥ 20 are reported. (Abbreviations: IPA=Ingenuity Pathway Analysis)

Table S6: Additional Information on Upstream Regulators From IPA.*

Upstream Regulator	Molecule Type	z-score	p-value	Target Molecules
TNF	cytokine	1.918	0.00442	CCR8, EGR2, G0S2, MEOX1, PHGDH, PRTN3, PTGS2, RETN, SLC7A8, SYNPO, TIMD4
CREB1	transcription regulator	1.397	0.0114	COCH, EGR2, OMG, PTGS2, SORCS3
PTEN	phosphatase	0.206	0.00627	EGR2, G0S2, MEOX1, PHGDH, PTGS2, SLC7A8
MRTFB	transcription regulator	0.152	0.000687	G0S2, PRTN3, PTGS2, TRNP1

* Significant upstream regulators with a with a q-value<0.05 and an absolute z-score<0 are reported. (Abbreviations: IPA=Ingenuity Pathway Analysis)

Table S7: Patient Characteristics of the Validation Cohort.*

	Patients with IA (n=6)	Patients without IA (n=7)	p-value
**Age (years)** (mean+SE)	42.7±5.28	56.7±4.89	0.078
[Q2 (Q1/Q3)]	40 (34/50.5)	62 (50.5/62.5)	
**Sex**			
Female	66.67%	57.14%	1
**Current Smoker**			
Yes	50.00%	28.57%	0.592
**Comorbidities**			
Osteoarthritis	16.67%	42.86%	0.559
Diabetes mellitus	0.00%	14.29%	1
Heart disease	0.00%	28.57%	0.462
Hyperlipidemia	33.33%	28.57%	1
Hypertension	16.67%	28.57%	1
Stroke history	16.67%	28.57%	1

*Clinical characteristics of the randomly-assigned validation cohort. These clinical factors were retrieved from patients' medical records. With the exception of age, these datapoints were quantified as binary data points. Significant differences between each group were evaluated with a Student's t-test for continuous data (age) and a Fisher's exact test for categorical data (α=0.05). There was no statistically significant difference in age, sex, smoking, or comorbidities between the IA and control groups. (Abbreviations: IA=intracranial aneurysm, n=number, Q=quartile, SE=standard error)

Table S8: Aneurysm Characteristics of the Validation Cohort.*

Characteristic	Value
**Family history of IA (n/n_{total})**	0/6 (0%)
**Patients with Multiple IAs (n/n_{total})**	1/6 (16.7%)
**Size (mm) [mean (min/max)]**	6.4 (3/10)
**IA Location (n/n_{total})**	
ACom	1/7 (14.3%)
BA	1/7 (14.3%)
Cavernous ICA	1/7 (14.3%)
ICA	2/7 (28.6%)
MCA	1/7 (14.3%)
PCom/PCA	1/7 (14.3%)

*Aneurysm size ranged from 3 mm to 10 mm. The aneurysms were situated at various locations in the Circle of Willis, with most residing again at the internal carotid artery and its branches. None of the patients had a family history of the disease. (Abbreviations: ACom=anterior communicating artery, BA=basilar Artery, IA=intracranial aneurysm, ICA=internal carotid artery, MCA=middle cerebral artery, n=number, PCA=posterior cerebellar artery, PCom=posterior communicating artery)

Table S9: All Correlation Analysis Results.*

	Correlation with IA Size			Correlation with ISUIA		
**Gene**	**PCC**	**95% CI**	**p-val**	**PCC**	**95% CI**	**p-val**
ADAMTS17	0.429859	[0.05 – 0.7]	0.028398	-0.10813	[-0.48 – 0.29]	0.59903
ANKRD22	0.029435	[-0.36 – 0.41]	0.886498	-0.02247	[-0.41 – 0.37]	0.913244
ANKRD24	-0.16438	[-0.52 – 0.24]	0.422298	-0.14132	[-0.5 – 0.26]	0.491069
ANKRD34B	0.096854	[-0.3 – 0.47]	0.637868	0.468448	[0.1 – 0.72]	0.015792
ANO5	0.120798	[-0.28 – 0.49]	0.556653	0.297858	[-0.1 – 0.61]	0.139442
BOK	-0.23607	[-0.57 – 0.17]	0.245637	-0.29368	[-0.61 – 0.11]	0.14535
C7orf61	-0.0607	[-0.44 – 0.33]	0.768326	-0.14516	[-0.5 – 0.26]	0.479229
CACNA1I	0.063152	[-0.33 – 0.44]	0.759238	-0.29149	[-0.61 – 0.11]	0.148505
CCR8	0.460603	[0.09 – 0.72]	0.017886	0.186343	[-0.22 – 0.54]	0.362054
CDCP1	0.128491	[-0.27 – 0.49]	0.531606	-0.08465	[-0.46 – 0.31]	0.680977
CNTNAP1	0.059704	[-0.34 – 0.44]	0.772033	-0.17284	[-0.53 – 0.23]	0.398471
COCH	0.171721	[-0.23 – 0.52]	0.401581	0.03634	[-0.36 – 0.42]	0.860102
CXorf67	0.092204	[-0.31 – 0.46]	0.654164	-0.21743	[-0.56 – 0.19]	0.285985
DEFA4	-0.12537	[-0.49 – 0.28]	0.541692	-0.08517	[-0.46 – 0.31]	0.679126
EGR2	-0.11761	[-0.48 – 0.28]	0.567178	-0.08241	[-0.46 – 0.32]	0.689002
FAM71F2	-0.16984	[-0.52 – 0.23]	0.406836	-0.15872	[-0.51 – 0.24]	0.438662
FAM83D	0.021499	[-0.37 – 0.41]	0.916977	-0.13054	[-0.49 – 0.27]	0.525038
FMN1	0.007483	[-0.38 – 0.39]	0.971058	-0.14359	[-0.5 – 0.26]	0.484066
FOLR3	-0.05162	[-0.43 – 0.34]	0.802258	-0.14316	[-0.5 – 0.26]	0.485367
G0S2	-0.25827	[-0.59 – 0.14]	0.202695	-0.24751	[-0.58 – 0.15]	0.222825
HLA-DQB2	-0.05005	[-0.43 – 0.34]	0.808169	-0.33953	[-0.64 – 0.06]	0.0897
HSPA2	0.234463	[-0.17 – 0.57]	0.248953	0.210915	[-0.19 – 0.55]	0.301013
KCNG1	-0.0234	[-0.41 – 0.37]	0.909645	-0.20007	[-0.55 – 0.2]	0.327109
KIR3DL2	0.052643	[-0.34 – 0.43]	0.798413	0.197909	[-0.21 – 0.54]	0.332473
L1TD1	0.012884	[-0.38 – 0.4]	0.95019	0.354834	[-0.04 – 0.65]	0.075286
MEOX1	-0.06837	[-0.44 – 0.33]	0.739985	-0.291	[-0.61 – 0.11]	0.149225
MKRN3 †	0.558093	[0.22 – 0.78]	0.003049	0.301667	[-0.1 – 0.62]	0.134212
ODF3B	0.147954	[-0.25 – 0.51]	0.470724	0.408777	[0.03 – 0.69]	0.038133
OMG	-0.12107	[-0.49 – 0.28]	0.555759	-0.2099	[-0.55 – 0.19]	0.303409
OR2AK2	-0.13769	[-0.5 – 0.26]	0.502385	-0.01057	[-0.4 – 0.38]	0.959122
PDZK1IP1	0.330016	[-0.07 – 0.64]	0.099661	0.12498	[-0.28 – 0.49]	0.542971
PHGDH †	-0.38706	[-0.67 – 0.]	0.050763	-0.45808	[-0.72 – -0.09]	0.018606
PHOSPHO1	-0.25931	[-0.59 – 0.14]	0.200831	-0.25582	[-0.59 – 0.15]	0.207172
PRTN3	-0.05629	[-0.43 – 0.34]	0.784752	0.103089	[-0.3 – 0.47]	0.616272
PTGS2	-0.20343	[-0.55 – 0.2]	0.318883	-0.14647	[-0.51 – 0.26]	0.475244
RAVER2	-0.12503	[-0.49 – 0.28]	0.542809	-0.06533	[-0.44 – 0.33]	0.751195
RETN	-0.16844	[-0.52 – 0.23]	0.410778	-0.10162	[-0.47 – 0.3]	0.62132
RP11-231C14.4	0.006594	[-0.38 – 0.39]	0.974496	-0.01111	[-0.4 – 0.38]	0.957034
SAMD14	0.264546	[-0.14 – 0.59]	0.191554	0.269657	[-0.13 – 0.59]	0.182795

SCARF2	-0.07195	[-0.45 – 0.32]	0.726883	-0.07383	[-0.45 – 0.32]	0.720014
SDC3	0.162637	[-0.24 – 0.52]	0.427306	0.328102	[-0.07 – 0.63]	0.101759
SHISA8	0.283847	[-0.12 – 0.6]	0.159944	0.371597	[-0.02 – 0.66]	0.061601
SLC7A8	-0.25557	[-0.59 – 0.15]	0.207618	-0.1871	[-0.54 – 0.22]	0.360073
SMN1	-0.02562	[-0.41 – 0.37]	0.90113	-0.26629	[-0.59 – 0.13]	0.188534
SORCS3	-0.09719	[-0.47 – 0.3]	0.636709	-0.25039	[-0.58 – 0.15]	0.217303
SSTR3	0.020817	[-0.37 – 0.4]	0.919603	-0.25508	[-0.58 – 0.15]	0.208524
SYNPO	0.119444	[-0.28 – 0.48]	0.561119	-0.21102	[-0.55 – 0.19]	0.300774
TIMD4 †	-0.28971	[-0.61 – 0.11]	0.151117	-0.49403	[-0.74 – -0.13]	0.010313
TNNT1	0.157598	[-0.24 – 0.51]	0.441954	0.027172	[-0.36 – 0.41]	0.895176
TRIM7 †	0.265114	[-0.14 – 0.59]	0.190567	0.399163	[0.01 – 0.68]	0.043374
TRNP1	-0.22145	[-0.56 – 0.18]	0.276947	-0.04795	[-0.43 – 0.35]	0.816073
TRPV4	0.036878	[-0.36 – 0.42]	0.858054	0.497969	[0.14 – 0.74]	0.009631
UTS2	0.237144	[-0.17 – 0.57]	0.243427	-0.01688	[-0.4 – 0.37]	0.934775
ZNF135	-0.08776	[-0.46 – 0.31]	0.669886	-0.36532	[-0.66 – 0.03]	0.066479

* Genes with an absolute PCC>0.25 in both groups are indicated by "†". (Abbreviations: CI=confidence interval, PCC=Pearson correlation coefficient)

Supplemental Figures

Figure S1

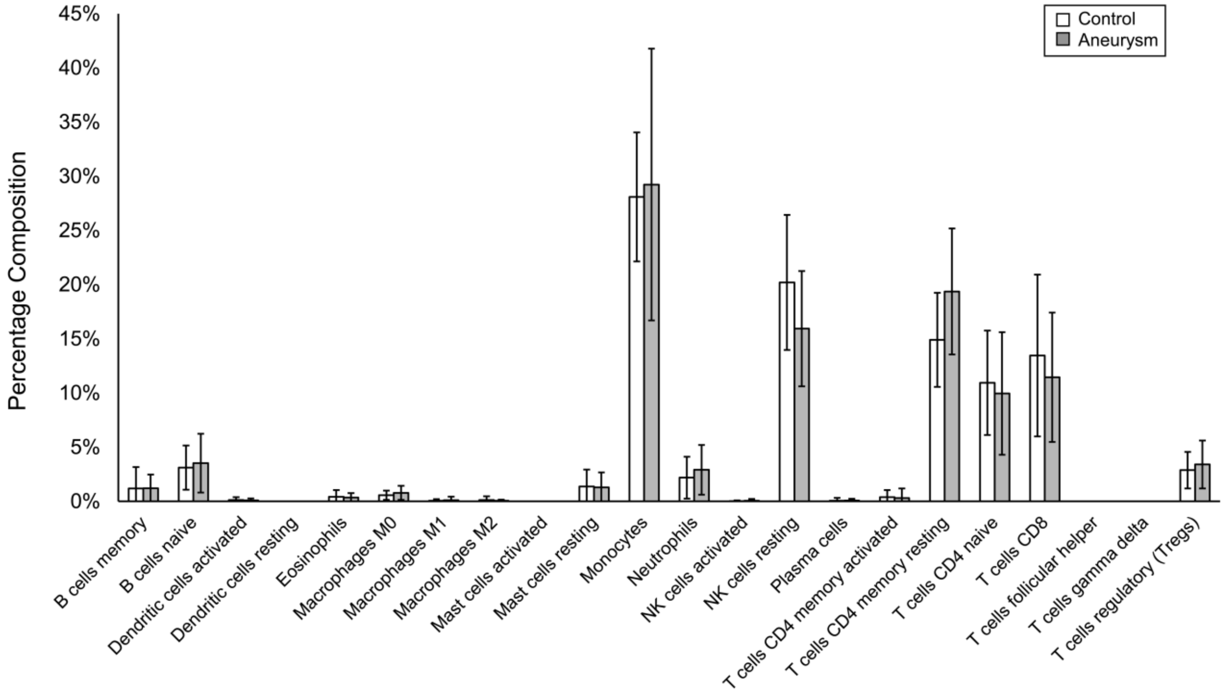

Figure S1: Distribution of cell types in isolated PBMCs of both groups computed by CIBERSORT. In the discovery cohort, there were no statistically significant differences between cell type proportions in the aneurysm (grey) and control groups (white). All comparisons had a false discovery rate-corrected q-value>0.05. In both groups, monocytes comprise majority of cells, followed by NK cells and several T cell populations.

Figure S2

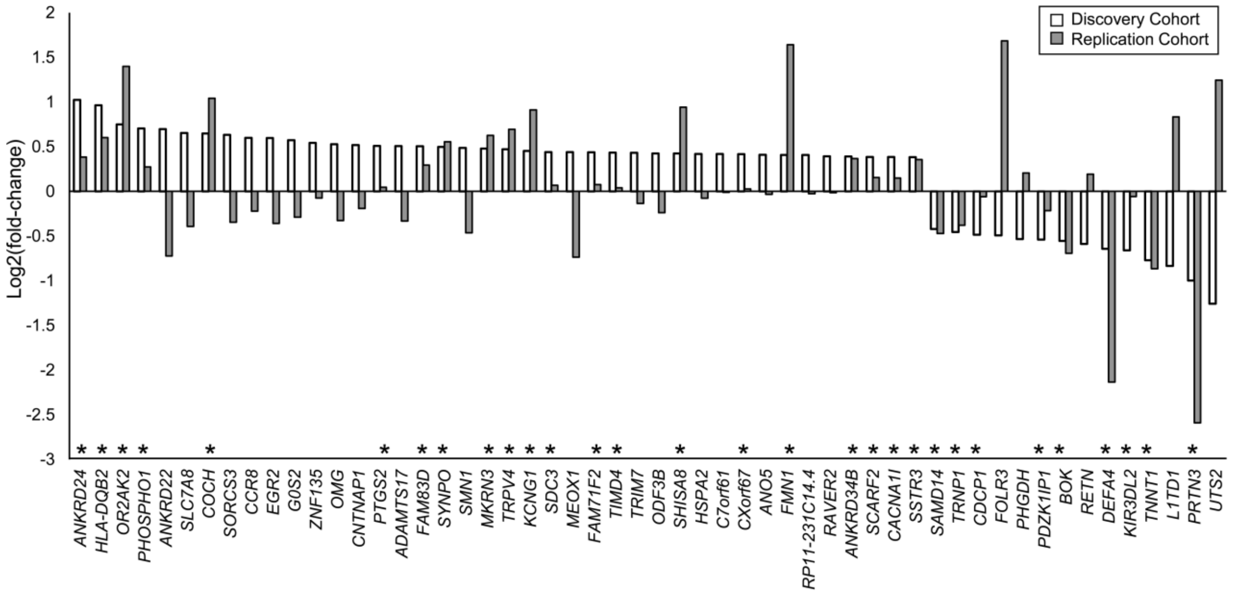

Figure S2: Comparison in fold-change of differentially expressed gene in the discovery cohort compared to the validation cohort. The 54 genes are presented in order of decreasing fold-change in the in the discovery cohort (white). A majority of the genes (30/54) have the same direction change in expression in the replication cohort (grey). These genes are indicated by the “*”.
